# The Known and Unknown About Female Reproductive Tract Mucus Rheological Properties

**DOI:** 10.1002/bies.70002

**Published:** 2025-03-22

**Authors:** Luke Achinger, Derek F. Kluczynski, Abigail Gladwell, Holly Heck, Faith Zhang, Ethan Good, Alexis Waggoner, Mykala Reinhart, Megan Good, Dawson Moore, Dennis Filatoff, Supriya Dhar, Elisa Nigro, Lucas Flanagan, Sunny Yadav, Trinity Williams, Aniruddha Ray, Tariq A. Shah, Matthew W. Liberatore, Tomer Avidor‐Reiss

**Affiliations:** ^1^ Department of Biological Sciences College of Natural Sciences and Mathematics University of Toledo Toledo Ohio USA; ^2^ Department of Physics and Astronomy College of Natural Sciences and Mathematics University of Toledo Toledo Ohio USA; ^3^ Department of Urology College of Medicine and Life Sciences University of Toledo Toledo Ohio USA; ^4^ Department of Chemical Engineering College of Engineering University of Toledo Toledo Ohio USA

**Keywords:** cervix, female reproductive tract, mucus, uterus, viscosity

## Abstract

Spermatozoa reach the fallopian tube during ovulation by traveling through the female reproductive tract mucus. This non‐Newtonian viscoelastic medium facilitates spermatozoon movement to accomplish fertilization or, in some cases, blocks spermatozoon movement, leading to infertility. While rheological properties are known to affect spermatozoon motility with in vitro models using synthetic polymers, their precise effects in vivo are understudied. This paper reviews the rheological measurements of reproductive tract mucus during ovulation in humans and model animals, focusing on viscosity and its potential effect on spermatozoa. Mucus viscosity in the female reproductive tract's different compartments is poorly understood. While information on this subject is incomplete, most mammals appear to have a viscosity decrease along their female reproductive tracts. Based on this sparse information, we hypothesize that viscosity changes in female reproductive tracts may guide spermatozoa to eggs, a novel concept that could improve our understanding of reproductive biology.

## Graphical abstract

Mucus viscosity changes as spermatozoa travel through the female reproductive tract to the egg in humans, bovines, mice, and other mammals. These changes may aid in spermatozoa navigation. However, limited information is available on the viscoelastic properties of female reproductive tract mucus, and consistent measurement techniques are lacking.

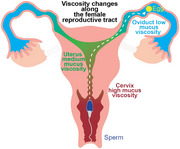

## 1 Introduction

The spermatozoon must reach and fertilize the egg to develop a new offspring. In humans and other species with internal fertilization (sexual reproduction where a spermatozoon fertilizes the egg inside the female body), spermatozoa travel through the female reproductive tract to reach the egg in a liquid‐like substance called mucus [1]. Mucus properties affect spermatozoa swimming patterns and are crucial in understanding how spermatozoa travel to the egg [2]. Indeed, spermatozoa movement models have been developed, relying on rheological information [3, 4, 5]. Female reproductive tract mucus is a complex gel (aka biphasic gel) with many properties that affect the success of the spermatozoon's journey along its path and up to penetrating the egg during fertilization. This complexity includes water, ion, and protein composition differences throughout the menstrual cycle [6] and passive defense against foreign cells [7]. Here, we focus on rheological properties (the ability of mucus to flow or be deformed). Rheologically, mucus is a non‐Newtonian viscoelastic, heterogeneous material with properties that vary depending on the species, the stage in the menstrual cycle, and the female reproductive tract compartment [8]. Viscoelasticity is a property that simultaneously describes a substance's viscous and elastic properties, as these two properties are often interconnected. Of these rheological characteristics, viscosity has been studied the most. According to Soulsbury and Humphries, “viscosity is a fundamental driver in changing spermatozoon structure and, in turn, plays a vital role in shaping the biomechanical movement of the sperm” [9, p. 11]. Furthermore, Zimmer and Riffell suggested that fluid shear rates may act as an evolutionary pressure on gamete morphology and fertilization rates [10]. We will, therefore, focus on mucus viscosity and briefly address viscoelasticity.

Three main factors undermine the current understanding of female reproductive tract viscosity. First, there are conflicting reports in the literature. For example, Suarez and DeMott [11] state that spermatozoon travels through “viscous oviduct fluids.” Similarly, Miller, in his recent review, suggests, “uterotubal junction secretion appears viscoelastic due to the abundant mucus” [12, p. 307]. Yet, others say that the oviduct mucus has a variable consistency based on the female's stage in menstruation [13]. Second, obtaining and measuring female reproductive tract mucus poses technological and ethical challenges. Finally, studying viscosity requires bridging distant biological disciplines using highly specialized terminology and instruments, such as cell biology and biophysics. This distance creates a significant barrier that hinders progress. Therefore, this review provides a background to clarify the confusion, promotes cross‐discipline discussions, and proposes a path for future research.

Mucus is a viscoelastic gel produced by goblet epithelial cells in the female reproductive tract [14, 15, 16, 17, 18]. It has two major components as follows: a gel made of mucin glycoproteins and water‐soluble components such as ions, lipids, and various proteins [19].

Mucus's rheological characteristics, namely its viscoelastic properties, hinge on mucins, a family of polymer glycoproteins with gel‐forming properties. Mucins are categorized into the following two distinct types: membrane‐bound mucins (e.g., MUC1, MUC3, MUC4), which typically reside on cell surfaces and participate in cellular signaling and adhesion, and secreted mucins (e.g., MUC5AC, MUC5B, MUC7), which are released into the mucus and contribute to its structural integrity and functionality [20]. MUC4 and MUC5B are the predominant mucins expressed in the female reproductive tract, with MUC1, MUC5AC, and MUC6 being found in smaller amounts. Most of these are found in the cervix, but some are also expressed along the other compartments of the female reproductive tract [21, 22]. Interestingly, mucin viscosity can be affected by bicarbonate concentration, a substance released by the female reproductive tract [23].

The composition of female reproductive tract mucus changes with the progression of the menstrual cycle [24, 25]. While much is known about cervical mucus changes, little is known about uterine and oviduct mucus changes during the menstrual cycle. The discernable change in mucus properties is its viscosity, which affects spermatozoan travel and is regulated by estradiol and progesterone levels. These two steroid hormones are antagonistic and affect cervical mucus water, the primary cervical mucin glycoproteins MUC5B, electrolytes, and other protein concentrations [26]. As estradiol levels build during the follicular phase and peak at ovulation, specific glands in the cervix named L and S crypts start to secrete distinct mucus, increasing cervical mucus water concentration (to 93%–98%), along with increased MUC5B concentration [26, 27, 28]. After ovulation, in the luteal phase, a decrease in estradiol and an increase in progesterone cause water content and MUC5B concentration to decrease. These hormonal changes in the luteal phase coincide with high‐viscosity cervical mucus (four to seven times as viscous as ovulation cervical mucus), which inhibits spermatozoon travel [6, 24, 29].

Here, we summarize the known and unknown rheological properties of female reproductive tract mucus viscosity, mainly in four species as follows: humans, as this knowledge will help in diagnosing and treating infertility; bovines, as they are a standard model for studying female reproductive tract mucus and spermatozoa motility, sheep as their mucus viscosity is studied as a cause for infertility, and mice, since they are the most common genetic mammalian model system. However, mice's spermatozoa and female reproductive tract differ remarkably from humans and bovines and lack a glandular cervix [30].

Our main findings include the need for more quantitative data on mucus viscosity and rheological properties across species and female reproductive tract compartments, particularly during ovulation. Based on the limited information in the literature, we propose that the viscosity changes dramatically along the mammalian female reproductive tract, and these viscosity differences are essential in guiding spermatozoa to the egg.

## 2 Viscosity Parameters

Two rheological properties—viscosity and elasticity—determine spermatozoon behavior in female reproductive tract mucus [31]. Of the two, viscosity has been more intensively studied, and our discussion will focus on it.

*Viscosity* (aka dynamic viscosity in non‐Newtonian fluids) determines how a fluid, such as water or mucus, is resistant to flow and is measured in Pascal‐seconds (Pa·s) or equivalent units (Table 1). This measure is helpful for simple Newtonian fluids that maintain the same viscosity when measured at different shear rates. For example, water has a viscosity of 1 mPa·s at 20°C while honey has a viscosity of 2000–10,000 mPa·s at 20°C; mucus viscosity is usually between these values [32, 33, 34].

**TABLE 1:** Summary of SI parameters used to quantify rheological properties.

Parameter		Unit	Description
Viscosity		Pa · s	Describes the ability of a substance to resist flow at different temperatures.
Viscosity	Consistency index (*K*)	Pa · s*^n^*	Describes the resistance to the flow of non‐Newtonian fluids. Lower “*K*” value, substance is less viscous. Greater “*K*” value, substance is more viscous.
Viscoelasticity	Loss modulus (*G*″)	Pa	Describes the mechanical energy lost when a substance is deformed.
	Storage modulus (*G*′)	Pa	Describes the ability of a substance to recover its original shape after stress‐induced deformation.
	*G*″/*G*′ ratio (tan(𝛿))	N/A	Determines whether a substance is more viscous, viscoelastic, or elastic: If *G*″/*G*′ > 1, then the substance is more viscous. If *G*″/*G*′ = 1, then the substance is viscoelastic. If *G*″/*G*′ < 1, then the substance is more elastic.

The viscosity of more complex materials, such as the non‐Newtonian mucus of the female reproductive tract, can change dramatically with shear rate (varying flow speed spatially) at a constant temperature, and hence viscosity has varying values. For many macromolecular fluids, viscosity can be modeled as a power law where *τ* = *Kγ^n^* [29]. Tau (*τ*) is the shear stress (Pascals), Kappa is the consistency index (Pa·s^n^), gamma is the shear rate (1/s), and *n* is the flow behavior index (dimensionless). *K* and *n* are model parameters determined by fitting viscosity measurements. When *n* is lower than 1, fluid behavior is shear‐thinning (less viscous with the increasing flow); when *n* equals 1, fluid behavior is Newtonian; and when *n* is greater than 1, fluid behavior is shear‐thickening (more viscous with the increasing flow).

Mucus is viscoelastic *and is thus both viscous and elastic*. Viscoelasticity measurements capture the viscous and elastic contributions to viscosity using an oscillatory shear flow. Specifically, the loss modulus or viscous modulus (*G*″) and the storage modulus or elastic modulus (*G*′) quantify viscoelasticity. The viscous modulus (*G*″) measures the energy dissipated in a substance. The elastic modulus (*G*′) measures a substance's ability to regain its original shape when deformed. The viscous (*G*″) and elastic (*G*′) moduli are used to calculate a ratio of the viscoelastic properties (i.e., *G*″/*G*′ ratio) [35]. If G″ is much bigger than *G*′ (i.e., *G*″/*G*′>>>1), mucus behaves more liquid‐like. If *G*″ and *G*′ are equal, or near 1 (i.e., *G*″/*G*′ = 0.1–10), mucus is viscoelastic, behaving with a mixture of solid and liquid‐like properties. If *G*″ is much less than *G*′, (i.e., *G*″/*G*′<<<1), mucus behaves more elastic than viscous and is more solid or gel‐like.

Note that in our review, we will use SI units of viscosity (mPa·s), consistency index (Pa·s^n^), and *G*″ and *G*′ (Pa) (Table 1). However, the literature that studies female reproductive tract mucus uses variable units, such as Newton‐second/meter^2^ (N·s/m^2^), Poise (P), Centipoise (cP), and Dyn·s/cm^2^.

## 3 The Female Reproductive Tract Challenges Spermatozoa Travel

**FIGURE 1:**
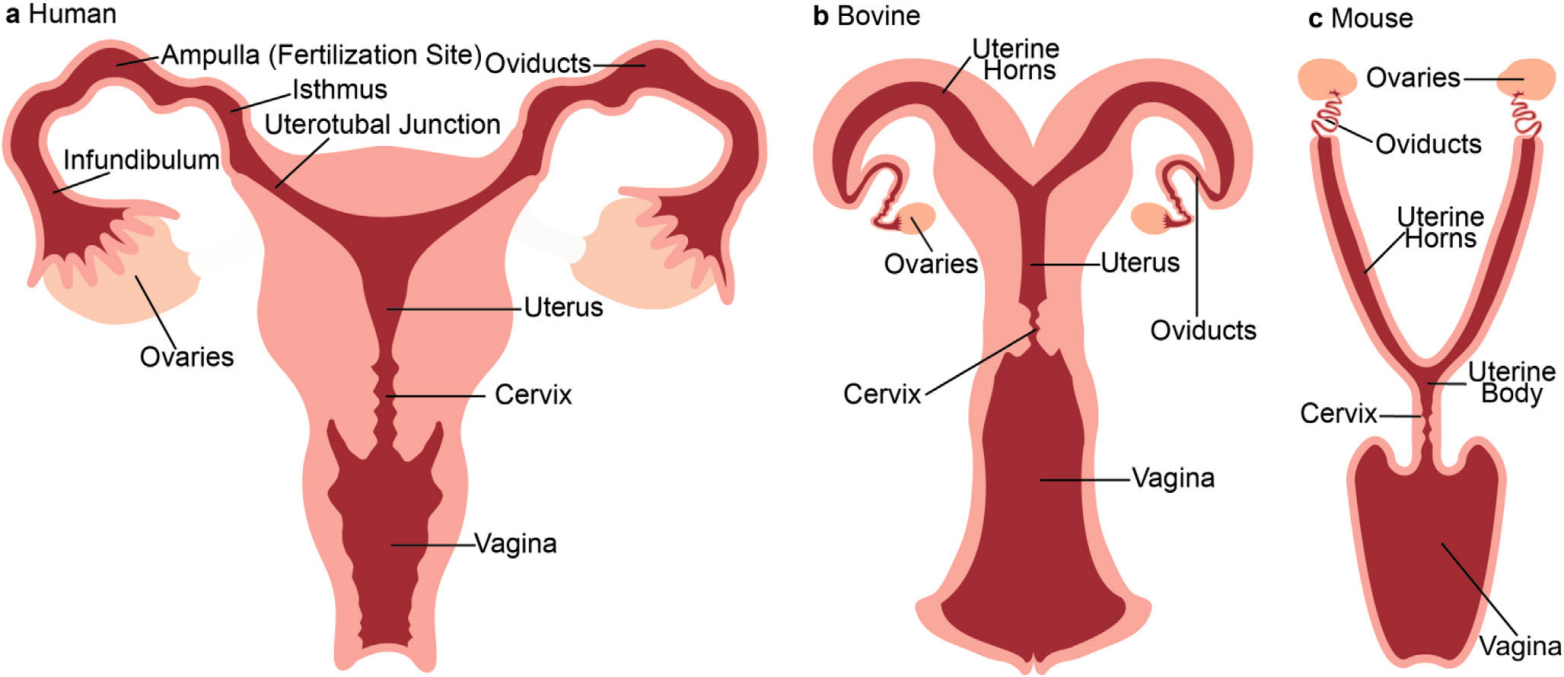
Mammalian female reproductive tracts exhibit similar anatomy with species‐specific adaptions. (a) Human, (b) bovine, and (c) mouse. The female reproductive tract mucus, secreted by goblet cells, regulates spermatozoa travel by varying estradiol and progesterone levels. Cervices crypts are shown as zig‐zag lines.

The female reproductive tract, from external to internal, consists of the vagina, cervix, uterus, uterotubal junction, and oviducts (Figure 1). The *vagina* is an acidic muscular tube where spermatozoa are deposited in most mammals, including humans, bovines, and mice. It is thought that human spermatozoa quickly migrate to the cervix to avoid the vagina's acidic environment; However, semen may naturally neutralize this acidity [36].

The crypt‐containing *cervix* is the first physical barrier to spermatozoa traveling through the female reproductive tract [37]. Cervical mucus controls permeability to the female reproductive tract, and its makeup changes throughout the menstrual cycle, especially during ovulation [24, 25]. One hypothesis is that highly viscous mucus plays a role in infertility, but very little is known about this topic [38]. The cervix and its mucus composition are among the most studied compartments of the tracts and across species.

The *uterus* is a muscular pear‐shaped compartment that develops the embryo and provides smooth muscle contractions to aid in childbirth [39]. Human uterine peristaltic contractions aid spermatozoon movement through the uterus [40]. These contractions increase in intensity and frequency as the follicular phase progresses (Days 1–14 of the human menstrual cycle) [40]. The uterus has species‐specific morphology: the human uterus is one large cavity (Figure 1a), the bovine uterus is partially split into two uterine horns (Figure 1b), and the mouse uterus splits completely into two long uterine horns (Figure 1c). It is important to note that, unlike in most depictions of the human uterus, there is little space in the uterus during the spermatozoon's journey to the egg, but rather, the walls of the uterus are compressed against each other with a narrow mucus‐filled space [41].

The *uterotubal junction* is the connection between the uterus and the oviduct and is narrowed and constricted compared to the uterus and isthmus. At least in cows, mice, sheep, and pigs, the uterotubal junction acts as the second physical barrier to spermatozoa travel to the egg, with most spermatozoa not making it to the other side. It is unknown if human female reproductive tracts follow this pattern. Additionally, it is unknown if the junction actively selects for normal motility spermatozoa [42]. This narrow lumen also presents a mucosal challenge to spermatozoa [43].

The *oviduct* acts as a passageway for the egg between the ovary and the uterus and is the last part of the spermatozoon's journey to the egg. The oviduct contains the isthmus, the ampulla, and infundibulum. In bovines, the isthmus is a spermatozoon reservoir, and spermatozoa are bound to its epithelial walls. The spermatozoa are gradually released towards the ampulla as ovulation nears [44]. The ampulla, a flask‐like location of the oviducts, is the site of fertilization in all mammalian species [45, 46]. In bovine and mice, the oviduct is a smaller and coiled organ that comes off the uterine horns.

## 4 The Female Reproductive Tract Mucus Changes During the Menstrual/Estrous Cycle

The menstrual cycle is divided into two phases, separated by ovulation and regulated by distinct hypothalamic hormones that affect the cervical mucus to select or exclude spermatozoa from entry into the female reproductive tract [47]. The *follicular phase* occurs approximately on Days 1–14 of the human menstrual cycle. It is induced by high estradiol (17‐beta‐estradiol) levels to create an environment that helps incoming spermatozoa travel by causing the cervical mucus to become abundant, watery, and stretchy [47]. *Ovulation* occurs approximately on Day 14 of the human menstrual cycle, separating the two phases. Changes to the cervix continue, further increasing the watery consistency of cervical mucus in preparation for possible incoming spermatozoa [47]. By the end of ovulation, estradiol levels decrease. The *luteal phase* occurs approximately on Days 14–28 of the human menstrual cycle. It is induced by progesterone produced by the corpus luteum to prepare the endometrium for the potential implantation of the blastocyst. Since the fertilization period has passed, progesterone thickens the cervical mucus, making it viscous. However, if the blastocyst is not implanted in the lining of the endometrium, spermatozoa will disintegrate and wash out through the female reproductive tract. The corpus luteum will then regress, and estradiol and progesterone serum levels will decrease, causing normal menstruation [47]. At this point, the endometrial layer can no longer be maintained and begins to shed.

The estrous cycle is the cyclic pattern of ovarian activity in most nonprimate mammals [48]. The cycle is divided into four phases as follows: proestrus and estrus occur during the follicular phase, and metestrus and diestrus occur in the luteal phase. In bovine, the estrous cycle lasts between 18 and 24 days, 4 and 6 days in the follicular phase, and 14 and 18 days in the luteal phase [49]. Like humans, bovine cervical mucus has a much more drastic 1000‐fold viscosity difference between estrus (human ovulation equivalent) to midcycle (close to middle luteal phase) [50].

The mouse estrous cycle lasts 4–5 days [51]. Surprisingly, no quantitative information was found on mucus rheology change during this cycle, even though mice are a standard animal model. This may be due to their low mucus volume.

## 5 Mucus Viscosity Is Measured by Macrorheology and Microrheology

Mucus viscosity is mainly measured using two methods that differ in ease of use and sensitivity: macrorheology and microrheology.

*Macrorheology*, also known as bulk rheology, is typically used in engineering to study the large‐scale properties of liquids. This technique is also commonly used to study female reproductive tract mucus. Standard macrorheological techniques include cone and plate rheometer and capillary viscometer [52, 53]. The major advantage of macrorheometry is that it is commonly available. Its disadvantages include needing substantial amounts of mucus (hundreds of microliters), which is challenging to collect from human or bovine oviducts and smaller mammals like mice. Another disadvantage is that macrorheology measures the whole volume as homogeneous, where mucus composition may change in different microenvironments [52].

*Microrheology* defines mucus’ local mechanical properties and can be separated into passive and active techniques [52]. The passive techniques include tracking particles exhibiting Brownian motion with manipulations done by thermal energy fluctuations [54, 55], which is based on the fluctuation–dissipation theorem [56]. Active methods are more common, and they measure viscosity by manipulating the added particles by an external force, such as magnetic tweezers [55]. Another advantage of microrheometry is that it can precisely measure a sample's local properties using smaller sample sizes (less than 50 microliters) [52, 57]. Also, it minimizes the deformation of the mucus sample by using smaller probes and particles [58]. Microrheometry disadvantages include the possibility of rapid dehydration due to smaller sample sizes, which can significantly affect the rheological properties of mucus [52]. Additionally, microrheometry using magnetic spheres has measurement variation depending on sphere diameter and coating [59].

Both macrorheological and microrheological techniques require access to the mucus in the female reproductive tract, which is only sometimes possible, especially in humans, due to the high cost and the need for skilled gynecologists. However, attempts have been made to develop techniques to generate and measure shear wave propagation, such as transvaginal ultrasound vibro‐elastography (TUVE), that can potentially overcome this limitation [60].

## 6 Human Reproductive Tract Mucus Measurements Vary Based on Technique and Menstrual Phase

Quantitative information on human mucus viscosity is only available for the cervix and oviduct. Using a torsion rheometer, Everhardt et al. found cervical mucus to have a viscosity of 874 mPa·s at body temperature [53]. Clift and Hart used a capillary‐tube viscometer at room temperature and found the viscosity to be 2500 mPa·s [63]. Studies on human cervical mucus showed a 4–7‐fold difference between the maximal peak at the beginning of the follicular phase and a minimum value during ovulation [24, 63]. Wolf et al. found that human cervical mucus at 30 rads/s (temperature not specified in paper) had a *G*′ of 4000 mPa and *G*″ of 4000 mPa with a *G*″/*G*′ ratio of 1 (ovulation) or a G′ of 7000 mPa, and *G*″ of 12,500 mPa with a *G*″/*G*′ ratio of ∼1.8 (the start of the follicular phase) displaying viscoelastic behavior [24]. Zhang et al. [60] utilized a new technique to study viscosity and elasticity in vivo called TUVE. This technique focuses on measuring shear wave propagation in the human uterus, specifically wave speed dispersion and frequency. They measured uterine shear elasticity (μ1) at 46,630 Pa and uterine shear viscosity (μ2) at 9580 mPa·s [60].

## 7 Bovine Reproductive Tract Mucus Is More Viscous in the Cervix/Vagina

Rutllant et al. characterized the *K* value of vaginal fluid samples using a viscometer and categorized them as Newtonian or non‐Newtonian [66]. Six out of 41 vaginal fluid samples were Newtonian viscosity coefficient reported; the remaining 35 samples were non‐Newtonian. The reported mean *K* for the Newtonian samples was 2 ± 1 mPa·s*^n^* ^,^ while the mean *K* of non‐Newtonian samples was 630 ± 149 mPa·s*^n^*. The *K* of non‐Newtonian samples increased from 237 ± 84 mPa·s*^n^* in the middle of estrus to 944 ± 229 mPa·s*^n^* at the end of estrus. Lopez‐Gatius et al. found a similar consistency index (*K*) by collecting anterior vaginal fluid at the beginning of estrus, finding *K* to be 575 mPa·s*^n^* at hour zero of estrus (no viscosity coefficient reported). No studies characterized the consistency index of fluids in the bovine uterus or oviduct [67].

Schultz et al. described bovine uterine fluid viscosity throughout the menstrual cycle qualitatively [68]. They found that the uterine fluid viscosity did not increase during the menstrual cycle and that the greatest volumes of uterine fluid were collected early in the menstrual cycle (follicular phase). Another study found a relative comparison between bovine uterine fluid and bovine vaginal fluid, with uterine horn secretions ranging from 0.1 to 1.6 in relative viscosity units and the vaginal fluid measured at 24 in comparison, and the cervical mucus also being qualitatively viscous, suggesting that bovine mucus gets less viscous further into the tract [69 as cited in, 70].

## 8 A Viscosity Decrease Is Present in the Uterine and Oviductal Fluids of Mice and Peromyscus Mice

Limitations to mice as models for cervical mucus studies include the size of the cervical canal and the insufficient amounts of cervical mucus that do not allow for rheological studies [71]. The low cervical mucus levels are likely because mice do not possess cervical glands like humans and bovine species [30]. Mice instead produce vaginal mucus, which is secreted by the vaginal epithelium.

To study the viscosities of *uterine* fluids in mice, Miki and Clapham [33] collected the fluids after dissecting the uterine horns, oviduct, and ovary approximately 60–90 min after sexual intercourse during estrus. The uterine luminal fluid with accumulated oviductal mucus, cell debris, and ejaculated semen exhibited a viscosity of 81 ± 73 mPa·s. They studied the viscosity of the fluids using the rolling‐ball method at 22°C [33]. This method involves filling a rolling ball viscometer tube with a sample and measuring the time it takes to roll a fixed distance [72].

A preprint by Hook et al. used euthanized Peromyscus mice whose female reproductive tracts were dissected to obtain their uterine and oviductal fluid using a glass Pasteur tube, squeezing the fluid out of the tissue [65]. The viscosity was measured at 37°C by multiple particle tracking (a microrheological technique). In this study, the Peromyscus mice were separated into polyandrous and monogamous groups (each with three species) based on how each species mates. Interestingly, polyandrous species of Peromyscus mice had a much higher average viscosity in the uterus at about 1790 mPa·s (the individual values were: *P. maniculatus* 40 mPa·s, *P. leucopus* 8,800 mPa·s, and *P. gossypinus* 2 mPa·s) than the monogamous mice who had an average of about 310 mPa·s (the individual values were *P. califorornicus* 290 mPa·s, *P. eremicus* 4 mPa·s, and *P. polionotus* 350 mPa·s). This trend seems to be only valid for the averages, as the individual values for oviduct and uterine viscosities are different for each Peromyscus mouse rather than separated by polyandrous versus monogamous mating mice. This could mean that the individual viscosities are specific to each Peromyscus mouse and not dependent on whether the mice mate polyandrously or monogamously.

They also found that the polyandrous mice have lower viscosity in the oviducts at about 10 mPa·s (the individual values were *P. maniculatus* 20 mPa·s, *P. leucopus* 6 mPa·s, and *P. gossypinus* 1 mPa·s) compared to the oviduct viscosity of the monogamous mice, which is 680 mPa·s (the individual values were *P. califorornicus* 40 mPa·s, *P. eremicus* 250 mPa·s, and *P. polionotus* 860 mPa·s). Intriguingly, the viscosities of the female reproductive tract differ from one species to another, suggesting the coevolution of the mating system, spermatozoa competition, and female cryptic choice.

## 9 Sheep Have Low‐Viscosity Cervical Mucus

Sheep are commonly raised as farm animals worldwide. Still, artificial insemination is limited due to low pregnancy rates using frozen semen compared to fresh semen in sheep (40% compared to 60%, respectively) [73]. This difference is observed in selected sheep breeds, and this uniqueness is attributed to differences in the properties of the cervical mucus. As a result, the viscosity of female reproductive tract mucus in sheep has been studied. Richardson et al. [73] compared mucus rheology in two breeds (Belclare and Suffolk) with differences in spermatozoa penetration. They found that both the elastic modulus and the complex modulus were significantly higher in Suffolk sheep (elastic modulus is 0.8 Pa in Suffolk and 0.4 Pa in Belclare), meaning that cervical mucus from Suffolk sheep behaves more like a solid than mucus from Belclare sheep. However, they did not find a significant difference between viscous moduli (0.5 Pa in Suffolk and 0.3 Pa in Belclare).

A similar but more extensive study compared the viscosity of cervical mucus between different sheep breeds. Abril‐Perreño et al. tested viscosity by measuring the time it took for samples from different breeds to fill the chamber [74]. They found that, during the follicular phase, Belclare sheep had significantly more viscous cervical mucus viscosity than Suffolk, but interestingly, during the luteal phase, the two viscosities were similar. This emphasizes the importance of collecting mucus for rheological testing during similar menstrual cycle phases. It should also be noted that these results do not contradict the results of Richardson et al. [73], as Richardson et al. measured the viscous modulus, not the viscosity of their samples.

Many papers in the published literature measuring viscosity use either qualitative descriptions to categorize different viscosity levels [75, 76] or nonstandard measures of viscosity, such as timing how long a sample takes to fill a chamber of a specific size [77, 78]. This makes it challenging to compare female reproductive tract viscosity in sheep to that of other species. However, Lee et al. describe lamb cervical mucus viscosity in standard viscosity units [79]. They collected cervical mucus from younger than 1‐year lambs from an undisclosed breed and measured its viscosity using a cone and plate digital rheometer. Intriguingly, the viscosity was determined to be much waterier, at about 4 mPa·s, than that of human, bovine, and mouse cervical mucus.

## 10 The Viscosities of the Female Reproductive Tract Compartments of the Horse, Pig, and Rabbit

Little is known about the viscosities of female reproductive mucus in other species besides humans, bovines, and mice. However, attempts have been made to collect and test the viscosity of reproductive tract mucus in rodents and domesticated animals.

Witte et al. extracted horse uterine mucus from three horses by a salivate rod and used a cone and plate rheometer to calculate the viscosity [80]. The uterine mucus viscosity values were variable depending on the day in estrous over the 5‐day cycle studied. On Day 1 of estrous detection, the uterine mucus viscosity ranged from 13 to 357 mPa·s between the three control group horses studied. On Day 5, the uterine mucus viscosity ranged from 375 to 648 mPa·s. There was a notable change in the viscosity measurement in the three horses studied from Day 1 to 5 of estrous.

Gonzalez‐Abreu et al. extracted pig oviduct mucus and used a cone and plate rheometer to measure the viscosity [81]. The oviduct mucus viscosity was measured at the late follicular and early luteal phases. The oviduct late follicular phase mucus viscosity ranged from 35 mPa·s at 1/s to 10 mPa·s at a shear rate of 200/s. The oviduct early luteal phase mucus viscosity ranged from 100 mPa·s at 1/s to 10 mPa·s at a shear rate of 200/s. The viscosity of the late follicular phase is lower, suggesting that the mucus becomes less viscous as ovulation draws near and prepares for fertilization.

A female rabbit has no estrus cycle, and ovulation occurs only after mating [82]. To mimic an estrous cycle, Hamner and Fox administered estradiol or progesterone over 5–8 days, followed by surgical removal of the oviducts and collection of mucus, with viscosity measurement by a Cannon‐Manning semi‐micro viscometer [83]. The oviductal mucus viscosity in the estradiol‐ and progesterone‐administered samples was 1 mPa·s. This may suggest that rabbit oviductal mucus viscosity may not change significantly during ovulation.

It should be noted that most studies are conducted at temperatures between 25°C and 37°C. This temperature change results in less than a 10% viscosity change, making the differences between studied samples negligible relative to the order of magnitude observed along the female reproductive tract [84].

## 11 Hypothesis

Spermatozoa must pass large and varied paths to reach and fertilize the egg, and only a tiny fraction of the ejaculated spermatozoa reach the vicinity of the egg [85]. Spermatozoon motility and behavior are affected by mucus viscosity [86]. Viscosity plays a critical role in selecting the spermatozoon reaching the egg, and in pathological conditions, reducing obstacles to reaching the oocyte can lead to an increase in poor‐quality spermatozoa passing the cervix, resulting in subfertility or infertility [87]. Therefore, knowing the viscosity in the female tract around the time of ovulation is clinically critical.

Furthermore, mucus viscosity can play a critical role in postcopulatory sexual selection [88]. Postcopulatory sexual selection results in the co‐evolution of the male and female traits, impacting the reproductive strategies and formation of new species [89, 90, 91]. Therefore, knowing the viscosity of the female tract around the time of ovulation is scientifically critical.

**FIGURE 2:**
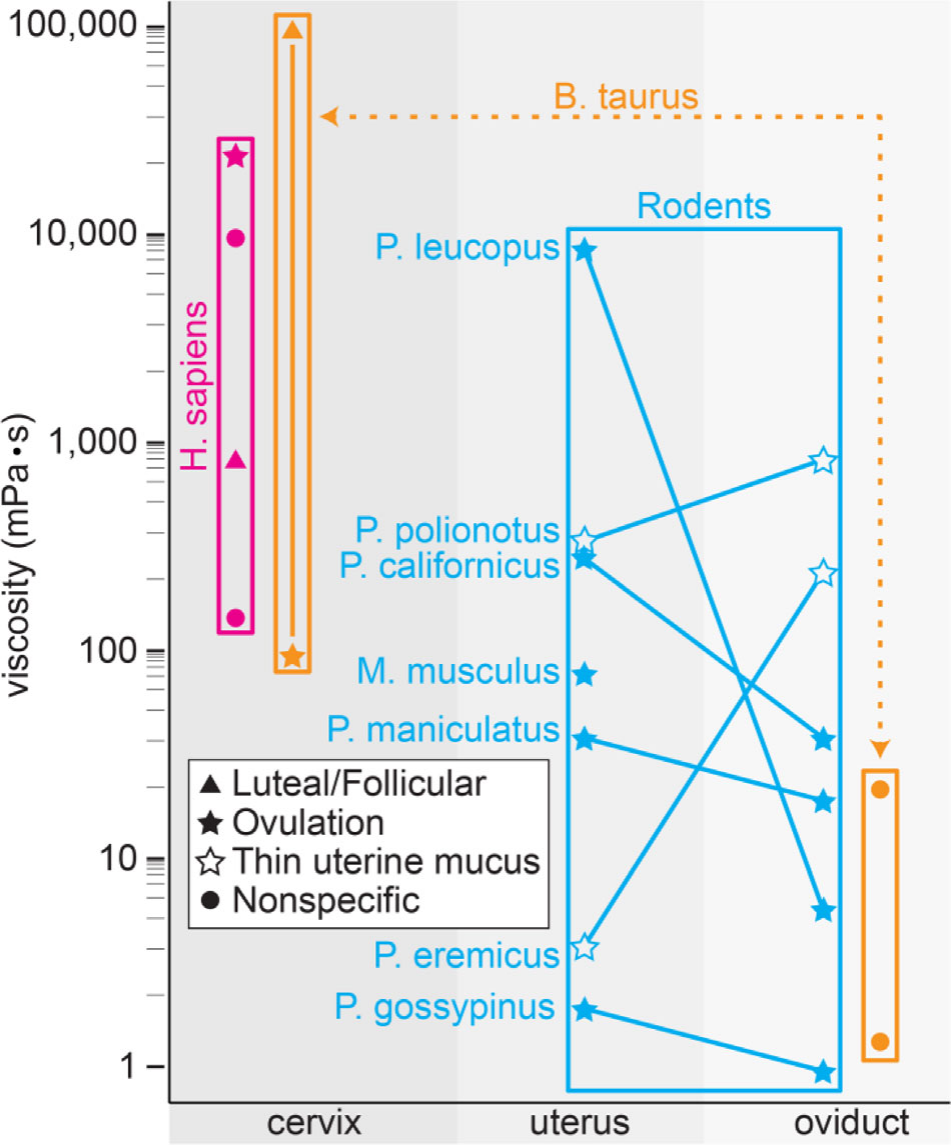
The literature has limited and variable information on the viscosity along the female tract. However, most studied species data support a viscosity decrease along the female reproductive tract. Human cervix viscosity (in magenta) from four papers is 200–50 Pa·s (within the magenta square). Bovine (in orange) cervix and oviduct data from four papers suggest a viscosity decrease along the female reproductive tract (orange square connected by a dotted line). Bovine data from the same publication are marked with a solid orange line. Seven species of rodents (in blue) report simultaneous viscosity analysis (line) in the uterus and oviduct. Five species had high‐to‐low viscosity changes (filled stars), and two had low to high (empty stars). Circles indicate nonspecific times in the menstrual cycle. Triangles indicate at or near the beginning of the follicular phase or the end or near the end of the luteal cycle. Stars indicate during ovulation. The human data were collected from the papers [53, 61–63]. The bovine data were collected from the papers [34, 50, 64]. The rodent data were collected from the papers [33, 65]. The measurements from papers [34, 50, 64] were taken at body temperature, whereas the other measurements were taken at room temperature.

**FIGURE 3:**
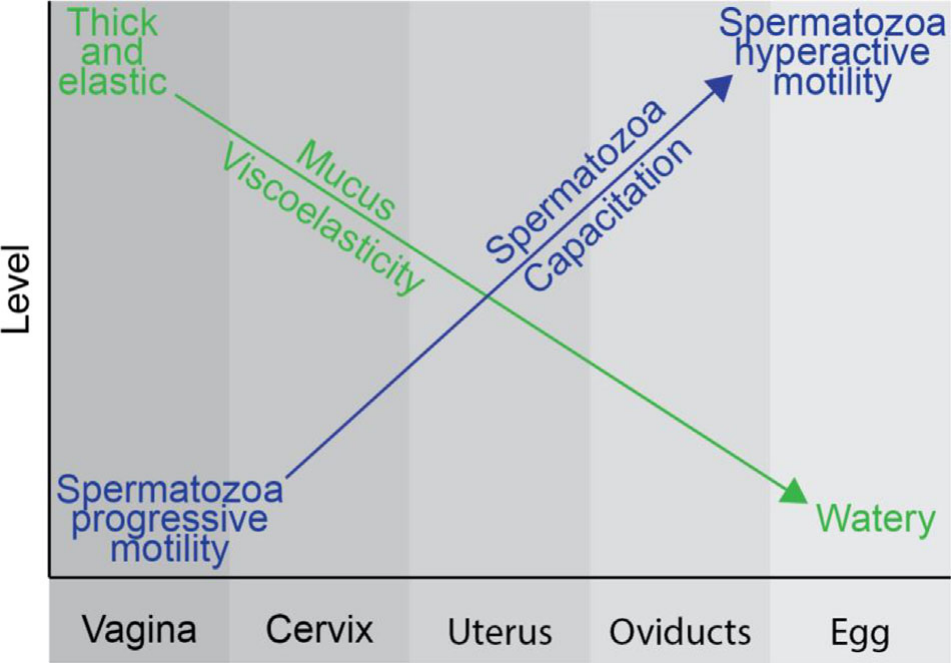
The hypothetical viscosity decreases along the female reproductive tract might interact with increasing spermatozoa capacitation along the female reproductive tract to facilitate spermatozoa's travel to the egg.

Summarizing the literature, we found scarce information, and the existing information has significant variations on the viscosity along the female tract (Figure 2). However, based on limited information, we hypothesize that viscosity changes exist in most mammalian female reproductive tracts, and they guide spermatozoa to eggs. In most mammals, these changes are from high viscosity in the cervix to medium viscosity in the uterus and low viscosity, similar to water, in the oviducts (Figure 3). Fragments of this decreasing viscosity were reported in humans, bovines, and mice. However, exceptions were described in some rodent species, specifically *P. eremicus* and *P. polionotus*, where viscosity increases rather than decreases. Additionally, past researchers have suggested, at least, high viscosity oviductal fluid that goes against the trend of decreasing viscosity along the female reproductive tract [11, 12]. This inverted viscosity gradient may act as an adaption that facilitates speciation [65].

These viscosity changes might facilitate spermatozoa's travel to the egg during estrus/ovulation, directing them to find the egg through another mechanism. The hypothetical viscosity decrease observed in most species may interact with other physiological changes along the female reproductive tract, such as the level of spermatozoon capacitation (Figure 3) [92]. Indeed, Sanchez‐Rodriguez et al. [92, p. 1] stated, “Increasing viscosity in the medium … result in additional time‐ and concentration‐dependent decrease in ATP in *M. spretus* and *M. spicilegus* under capacitating conditions” [92]. Additional changes along the female reproductive tract include temperature [93] and oxygen pressure [94].

The fluid temperature generally alters viscosity. Interestingly, temperature gradients within the female reproductive tract influence spermatozoabehavior [95]. Additionally, Foo describes a viscosity gradient existing in the female reproductive tract, yet he applies this concept to semen rather than to female reproductive tract mucus [96]. However, Hunter et al. interpret the initial findings as indicative of a viscosity gradient in the female reproductive tract [95].

## 12 The Knowledge Gap and How to Close It–Future Direction

The data on female reproductive tract mucus viscosity are fragmented due to two main factors. First, collecting mucus is easier in the cervix but difficult in the inner parts of the female reproductive tract. Second, the mucus volumes are usually larger in the cervix than in the inner parts of the female reproductive tract. Yet, it is unclear why uterine mucus data are more sparse, as even in mice at estrus post‐mating, an appreciable amount of uterine mucus can be recovered (22–87 mg) [33]. Another complexity is that uterine fluid may be contaminated by other liquids, such as oviductal mucus, cell debris, and ejaculated spermatozoa during mucus recovery, inflating the amount and changing its properties [33].

Currently, the rheological properties of cervical mucus are gathered via macrorheology and microrheology. A sufficient fluid volume must be present to run the samples through the current technology. While cervical mucus is abundant, the other compartments of the female reproductive tracts, especially the oviducts, do not provide the necessary volume to perform traditional rheology experiments. Other sensitive microrheology tools that measure the microliters of fluid are more applicable, such as some groups having used particle tracking software to estimate the rheological properties of the bovine oviductal fluid in microliter amounts [34, 97]. Unfortunately, with the nonuniformity of the field of mucus rheology, especially female reproductive tract mucus, spanning multiple decades, it could be that different rheological methods have varied outcomes. There may also be variability within the same methods as particles of different sizes measure different outcomes [52].

Interestingly, Zhang et al. [60] used TUVE to measure the viscoelasticity of human uterine mucus. Using an ultrasound at 100–300 Hz, they measured the wave speed dispersion of vibrations sent through the uterus at specific frequencies, and they used a mathematical model to determine the fluid's viscoelasticity. This method could potentially measure the viscoelasticity of female reproductive tracts in situ. The future direction involves developing more sensitive microrheometry tools to analyze minuscule volumes effectively.

Spermatozoon swimming patterns within different viscosities have been studied to some extent [86]. However, most of this research uses various techniques, preventing data comparison between studies and across species. Only recently has research been done on spermatozoa traveling through viscoelastic changes, and even then, it has only been done with synthetic polymers. A study by Tung et al. shows that spermatozoa cooperation increased in increasing viscoelastic mediums but not solely viscous media [31]. Additionally, Xiao et al. tested the concept of spermatozoon cooperation in human spermatozoa in vitro from a high‐viscosity to a low‐viscosity environment [98]. They found that human spermatozoon cooperation is lost when transitioning from the high‐viscosity to the low‐viscosity environment. More research is needed to test the various properties of spermatozoa across a viscoelastic change to see how spermatozoa react to the changing environment.

## 13 Spermatozoa Behave Differently in Different Viscosities

**FIGURE 4:**
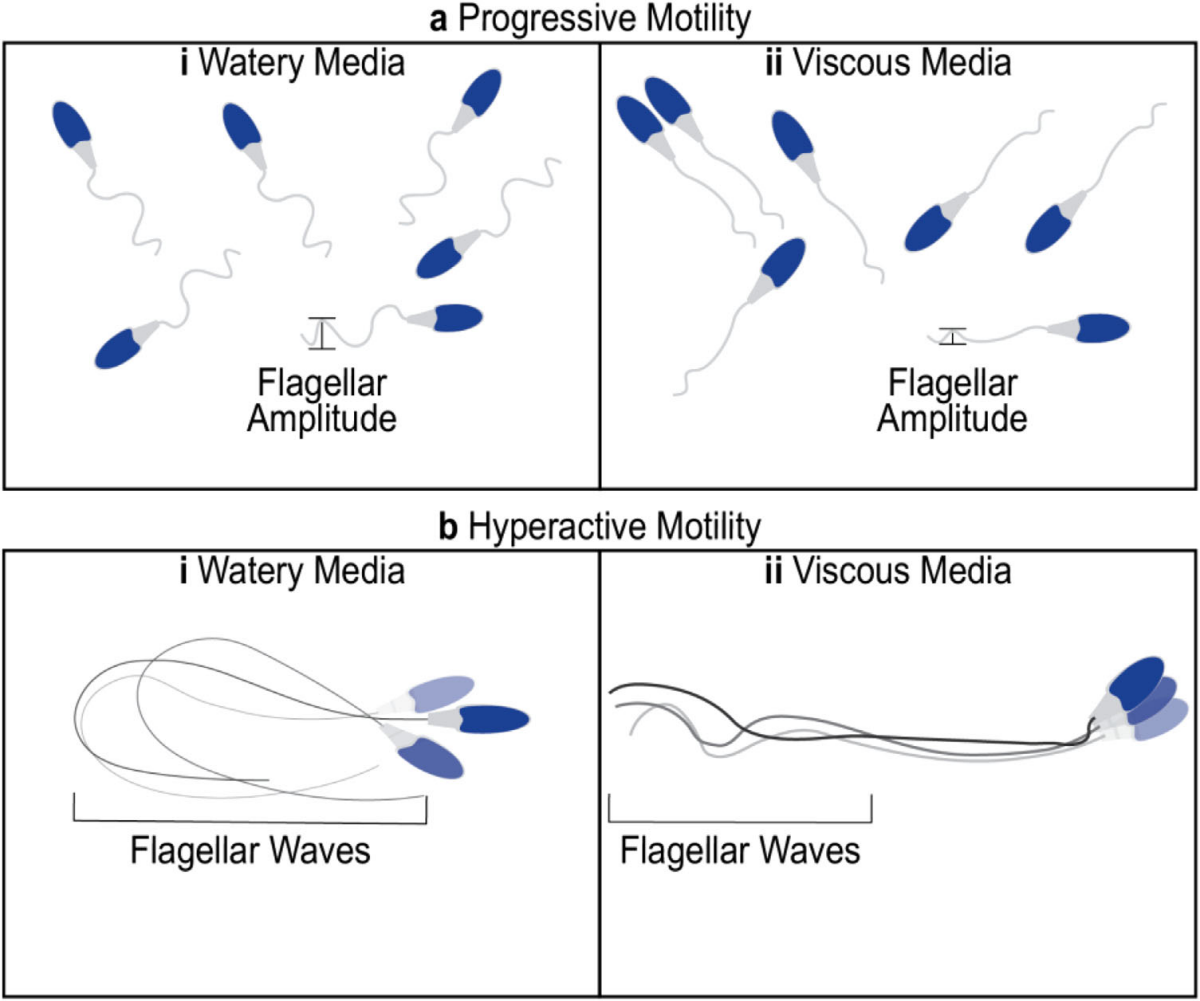
Spermatozoa motility in water and viscous media differs depending on whether it is progressive (a) or hyperactive (b) motility. Progressive motility spermatozoa in watery media (ai) have an increased flagellar amplitude than viscous media (aii). Hyperactivated spermatozoa in watery media exhibit large, whip‐like flagellar patterns (bi). In contrast, in viscous media, only the latter half of the principal piece and the entire endpiece move because of the lack of accessory structures in those parts (bii).

Spermatozoa must adapt to the changing viscosity in the female reproductive tract. While small molecules, like viruses, can diffuse freely in viscoelastic media in some environments, spermatozoa are too large to diffuse and must adapt their behavior to move progressively [52]. In general, spermatozoa flagella undergo waveform changes, like amplitude, frequency, wave speed, and wavelength, to adjust to different viscosities [34, 86]. Spermatozoa head rotation also changes when encountering various viscosity levels, dependent on capacitation factors [105]. Lastly, progressive (Figure 4a) and hyperactive (Figure 4b) motility also change depending on viscoelasticity [31, 106]. Capacitation factors resulting in progressive and hyperactivated motility may allow spermatozoa to travel successfully, depending on the mucus viscoelasticity at any one point throughout the female reproductive tract. For example, Smith et al. found differences in wave speeds, lengths, and frequencies of human spermatozoa in low and high viscosities in vitro [86]. From low to high viscosities mediums, spermatozoa rolling rates decreased from about 11 to 1.5 Hz, wave speeds decreased from 890 to 200 µm/s, wavelengths decreased from 39 to 18 µm, and wave frequency decreased from 23 to 11 Hz. Progression per beat, the distance a spermatozoon moves per beat cycle, increased with viscosity from a distance of 2.7–5.8 µm [86, 107]. However, Smith et al. observed no differences in progressive velocity in low and high viscosities of human spermatozoa (62 and 65 µm/s, respectively) [86]. Suarez and Dai found that hyperactivation enhances mouse spermatozoa capacity for penetrating viscoelastic media [106].

## 14 Implications for Fertility and Disease

Spermatozoa motility inside the female reproductive tract adapts to changing environments, most notably viscosity changes along the female reproductive tract (Figure 4). One of the main changes is the switch from a 3D movement to a 2D slithering and crawling movement along surfaces [99]. This implies that spermatthat cannot adapt to different environments have a decreased chance of reaching the egg and fertilizing it. Other adaptions include collective spermatozoa swimming, which improves fertility, which has been observed in bovine and mice spermatozoa [31, 100]. This cooperative swimming was hypothesized to allow for cell–cell communication and assist spermatozoa to travel through the female reproductive tract [31, 101]. Yet, it was suggested that high mucus viscosity could inhibit spermatozoa travel and thus decrease fertility [102].

However, there is a lack of knowledge about how changing viscosity in the female reproductive tract affects spermatozoa; Kirman‐Brown and Smith acknowledge this and call for more research [103]. Interestingly, they raised similar issues to those we raised in this article about the validity of previous work. They cautioned against throwing out all past studies and proposed to re‐analyze or repeat the work.

Clinically, there can be many reasons why mucus viscosity is affected, either directly or indirectly. Diseases that disrupt the female reproductive tract environment may cause hormonal, pH, or ionic changes that affect mucus viscosity. Additionally, scarring of cervical tissue from procedures or drugs (i.e., clomiphene citrate and propranolol) can reduce mucus production [104].

## 15 Conclusion

This review addresses a significant gap in the field of reproductive physiology. We aim to provide a quantitative understanding of the rheological properties of the female reproductive tract, a topic that has yet to be extensively explored. The lack of a standardized shear rate or frequency for measuring viscosity has led to varying results, even when the same sample is tested in different laboratories. Thus, comparing quantitative rheological properties between species and in various compartments of the female reproductive tract requires more experimentation. Based on this fragmented information, we hypothesize that changes in viscosity in female reproductive tracts may guide spermatozoa to eggs, a novel concept that could improve our understanding of reproductive biology. There is a pressing need for more comparative research on how spermatozoa traverse distinct species’ reproductive tracts during the biologically significant estrus period. It is essential to compare the female reproductive tracts of humans, bovines, and mice under identical conditions. This approach will enable us to understand better how viscosity influences spermatozoa as they journey through different species' reproductive tracts.

Similarly, it would be helpful to test how spermatozoa from different species navigate viscosity changes. A future goal is to understand the effects of viscoelastic changes on spermatozoa traveling through the female reproductive tract. Studying different compartments of the female reproductive tract is crucial as it allows us to understand each area's specific characteristics and functions. This knowledge can help researchers identify potential factors that may affect fertility, develop targeted treatments, and improve reproductive health outcomes for women. Two related questions are as follows: How does the viscoelastic decrease affect spermatozoa navigation?, and how does viscoelasticity change along the tract?

## Author Contributions

All authors read, edited, and approved the final manuscript. **Luke Achinger**: writing–original draft, writing–review and editing, visualization, supervision. **Derek F. Kluczynski**: writing–original draft, writing–review and editing, visualization. **Abigail Gladwell**: writing–original draft, visualization. **Holly Heck**: writing–original draft, visualization. **Faith Zhang**: writing–original draft. **Ethan Good**: writing–original draft. **Alexis Waggoner**: writing–original draft. **Mykala Reinhart**: writing–original draft. **Megan Good**: writing–original draft. **Dawson Moore**: writing–original draft. **Dennis Filatoff**: writing–original draft. **Supriya Dhar**: writing–original draft, visualization. **Elisa Nigro**: writing–original draft. **Lucas Flanagan**: writing–original draft, visualization. **Sunny Yadav**: writing–original draft. **Trinity Williams**: writing–original draft. **Aniruddha Ray**: resources, writing–review and editing. **Tariq A. Shah**: resources, writing–review and editing. **Matthew W. Liberatore**: resources, writing–review and editing. **Tomer Avidor‐Reiss**: conceptualization, writing–review and editing, supervision, funding acquisition.

## Conflicts of Interest

The authors declare no conflicts of interest.

## Data Availability

Data sharing is not applicable as no new datasets were generated.
